# Composition and RNA binding specificity of metazoan RNase MRP

**DOI:** 10.1093/nar/gkaf829

**Published:** 2025-08-27

**Authors:** Yuan Liu, Shiyang He, Kawon Pyo, Shanshan Zheng, Meijuan Chen, Bryony Braschi, Sihem Cheloufi, Nikolai Slavov, William F Marzluff, Jernej Murn

**Affiliations:** Department of Biochemistry, University of California, Riverside, CA 92521, United States; Center for RNA Biology and Medicine, University of California, Riverside, CA 92521, United States; Stem Cell Center, University of California, Riverside, CA 92521, United States; Department of Biochemistry, University of California, Riverside, CA 92521, United States; Center for RNA Biology and Medicine, University of California, Riverside, CA 92521, United States; Stem Cell Center, University of California, Riverside, CA 92521, United States; Department of Biochemistry, University of California, Riverside, CA 92521, United States; Departments of Bioengineering, Biology, Chemistry, and Chemical Biology, Single Cell Proteomics Center, and Barnett Institute, Northeastern University, Boston, MA 02115, United States; Department of Biochemistry, University of California, Riverside, CA 92521, United States; Stem Cell Center, University of California, Riverside, CA 92521, United States; HUGO Gene Nomenclature Committee, Department of Haematology, University of Cambridge School of Clinical Medicine, Cambridge, CB2 0AW, Cambridgeshire, United Kingdom; Department of Biochemistry, University of California, Riverside, CA 92521, United States; Center for RNA Biology and Medicine, University of California, Riverside, CA 92521, United States; Stem Cell Center, University of California, Riverside, CA 92521, United States; Departments of Bioengineering, Biology, Chemistry, and Chemical Biology, Single Cell Proteomics Center, and Barnett Institute, Northeastern University, Boston, MA 02115, United States; Integrated Program for Biological and Genome Sciences, University of North Carolina, Chapel Hill, NC 27599, United States; Department of Biochemistry and Biophysics, University of North Carolina, Chapel Hill, NC 27599, United States; Department of Biochemistry, University of California, Riverside, CA 92521, United States; Center for RNA Biology and Medicine, University of California, Riverside, CA 92521, United States; Stem Cell Center, University of California, Riverside, CA 92521, United States

## Abstract

Ribonuclease (RNase) MRP is a conserved RNA-based enzyme best known for its essential role in the maturation of ribosomal RNA (rRNA) in eukaryotes. However, the composition and RNA substrate specificity of this multisubunit ribonucleoprotein complex in higher eukaryotes remain a mystery. Here, we identify NEPRO and C18ORF21 (which we renamed RMP64 and RMP24, respectively) as constitutive subunits of metazoan RNase MRP. These proteins are unique to RNase MRP and absent from the closely related RNase P, which processes transfer RNA (tRNA) precursors and tRNA-like substrates. We find that RMP64 and RMP24 are integral subunits of RNase MRP, stabilize its catalytic RNA, and are required for rRNA maturation and cell proliferation. Leveraging these discoveries, we identify a broad suite of *in vivo* RNA-binding targets of each enzyme, including potential cleavage sites at nucleotide resolution. Our findings identify the first metazoan RNase MRP-specific protein subunits and define the RNA-targeting repertoire of this essential enzyme in mammalian cells.

## Graphical Abstract

**Graphical Abstract:**
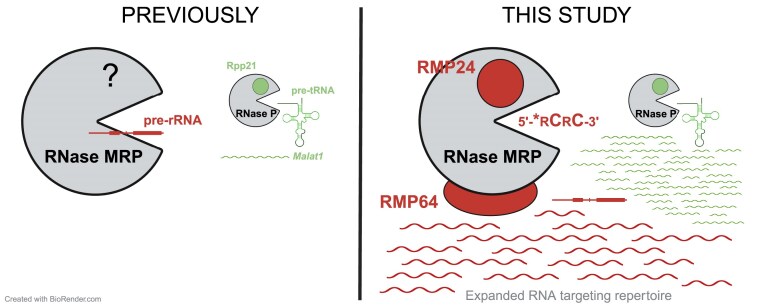

## Introduction

**Figure 1.**
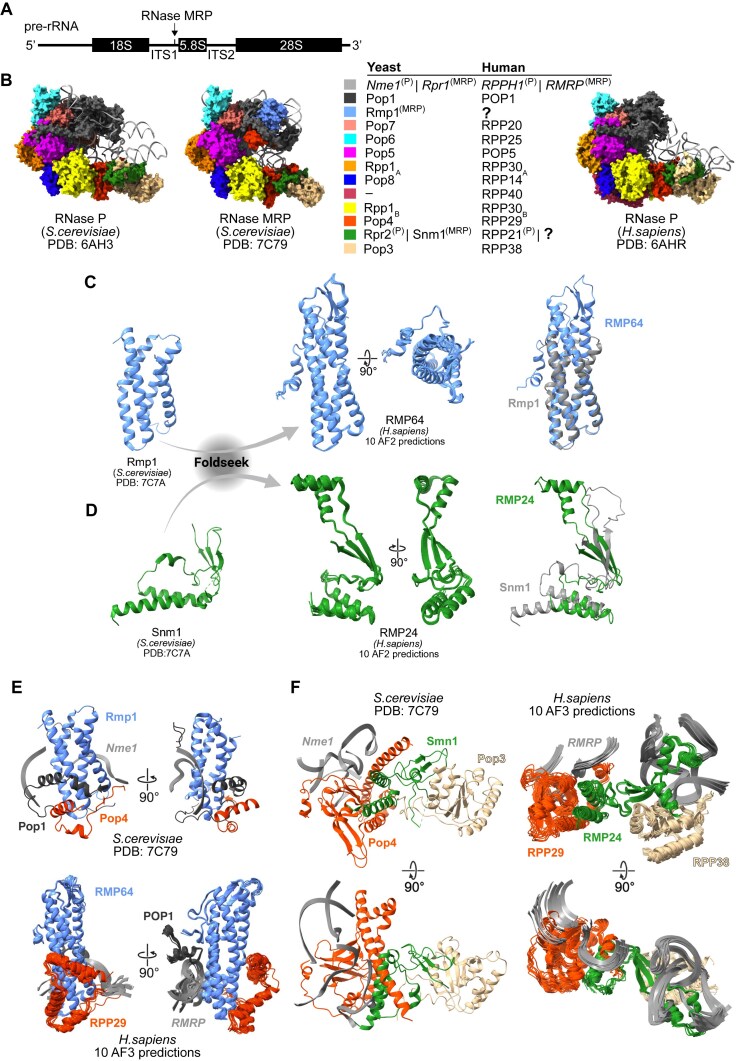
Structural homology identifies two novel candidate subunits of metazoan RNase MRP. (**A**) Schematic indicating the location of the annotated RNase MRP cleavage site within ITS1 of 47S pre-rRNA. (**B**) Cryogenic-electron microscopy (cryo-EM) structures of yeast RNase P (PDB: 6AH3, left), yeast RNase MRP (PDB: 7C79, middle), and human RNase P (PDB: 6AHR, right) along with a listing of the known RNase P and RNase MRP subunits in yeast and human. Labels (**P**) or (MRP) indicate subunits specific to RNase P or RNase MRP, respectively. (**C**,**D**) Foldseek identified human RMP64 and RMP24 as structural homologs of yeast Rmp1 and Snm1, respectively. Cryo-EM structures of Rmp1 (C) and Snm1 (**D**, both PDB: 7C7A) were used as queries. Superpositions of 10 AlphaFold2 (AF2) predictions of the converging segments of human RMP64 and RMP24 are shown. The same segments of each protein (one AF2 prediction) were also used for their alignments with the cryo-EM structures of Rmp1 and Snm1, as shown. (**E**) Interfaces of yeast Rmp1 interacting with Pop1, Pop4, and *Nme1* (PDB: 7C79, top) compared to 10 AlphaFold3 (AF3) predictions of human RMP64 interacting with POP1, RPP29, and *RMRP* (bottom). Ten AF3 predictions of human RNase MRP were generated using full-length sequences of all human subunits listed in panel (B), RMP64, and RMP24. The predictions were aligned on RMP64. Only the converged segments of RMP64 and its interacting subunits are shown. (**F**) As in panel (E) but showing interfaces of yeast Snm1 interacting with Pop4, Pop3, and *Nme1* (PDB: 7C79, left) compared to ten AF3 predictions of human RMP24 interacting with POP1, RPP29, and *RMRP* (right). The predictions of the human RNase MRP were aligned on RMP24, and only the converged segments of RMP24 and its interacting subunits are shown.

The maturation of 18S, 5.8S, and 28S ribosomal RNA (rRNA) begins with endonucleolytic cleavages of the polycistronic precursor rRNA (pre-rRNA) and continues with exonucleolytic trimming to produce the mature rRNAs essential for the formation of functional ribosomes [1]. Cleavage that splits pre-rRNA into parts destined for incorporation into the small or the large subunit of the human ribosome is mediated by a poorly understood, evolutionarily ancient RNA-based enzyme, RNase MRP (Fig. 1A) [1, 2]. This essential eukaryotic ribonucleoprotein (RNP) is known to consist of at least nine protein subunits (POP1, POP5, RPP14, RPP20, RPP25, RPP29, RPP30, RPP38, and RPP40) and a catalytic RNA, termed *RMRP* [3, 4]. While RNase MRP’s role in rRNA processing is well established [1, 5–7], its full composition, RNA substrate repertoire, and additional functions in mammalian cells remain incompletely understood [4, 8]. The essential role of RNase MRP in human cells is highlighted by mutations in the body or promoter region of *RMRP*, as well as in certain RNase MRP protein subunits, which cause various pleiotropic diseases, including cartilage-hair hypoplasia characterized by skeletal abnormalities, immune system deficiency, and increased cancer risk [9–17].

RNase MRP is structurally and evolutionarily related to another essential RNA-based enzyme, RNase P, which is required for the maturation of the 5′ end of precursor transfer RNA (pre-tRNA) in all three domains of life [4, 5]. Structural studies in yeast reveal that both RNPs use a similar catalytic core within the RNA moiety and share most protein subunits (Fig. 1B) [4, 7, 18]. However, several RNA elements and a handful of protein subunits distinguish RNase MRP from RNase P in yeast [7, 18]. These differences are thought to underlie the enzymes’ distinct modes of RNA substrate recognition: “measuring” the L-shaped pre-tRNA-like structures by RNase P and recognition of a short consensus sequence in single-stranded RNA substrates by yeast RNase MRP [7, 19–23]. Whereas one human RNase P-specific protein has been identified (RPP21; orthologous to Rpr2 in yeast) [24], no human RNase MRP-specific proteins were found, although two such proteins, Rmp1 and Snm1, exist in yeast (Fig. 1B) [4, 25, 26]. The apparent absence of RNase MRP-specific subunits has long limited our understanding of the enzyme’s unique features in higher eukaryotes, despite its essential role in rRNA processing and well-established evolutionary relationship to RNase P [3, 4].

A vexing question is about the sheer existence of RNase MRP in higher eukaryotes: why would a simple hydrolytic cleavage of one RNA substrate require a half-megadalton RNA-based catalyst in human cells? RNase MRP is thought to have emerged in eukaryotes as an RNase P-like enzyme to fulfill a new requirement for pre-rRNA processing [5]. This could justify its dedication to processing of pre-rRNA in the earliest eukaryotic organisms about two billion years ago [27]. However, it is difficult to envision that RNase MRP would sustain its single-substrate specificity in higher eukaryotes, especially considering that at least two additional RNase MRP substrates, *CLB2* and *CTS1* messenger RNAs (mRNAs), have been identified in yeast, and that several other site-specific pre-rRNA cleavages in mammalian cells are catalyzed by protein-only nucleases [28–30]. The persistence of RNase MRP conceivably reflects a need for processing of multiple RNA substrates, likely with some level of sequence specificity [19]. Defining these and the purpose of their processing by RNase MRP should help decipher the *in vivo* function of this RNP.

Here we use structural predictions to discover two RNase MRP-specific protein subunits in metazoans. We validate that RMP64 and C18ORF21 (recently renamed RMP64 and RMP24, see “Materials and methods” section) are unique subunits of mammalian RNase MRP via analyses of their protein–protein and protein–RNA interactions, critical roles in pre-rRNA processing, and requirement for cell proliferation. We leverage our discovery to distinguish RNase P from RNase MRP and define complete repertoires of their *in vivo* RNA targets, along with positions of putative cleavage sites at nucleotide resolution. These results shed light on the composition and RNA-binding specificity of metazoan RNase MRP.

## Materials and methods

### Cell culture and treatment

HEK293T cells were maintained in DMEM supplemented with 10% FBS (Cytiva, SH30910.03) and 100 U/ml Penicillin-Streptomycin (Gibco, 15140122) at 37°C and 5% CO_2_. mESC E14 cells were maintained in DMEM supplemented with 15% FBS (Cytiva, SH30910.03), 1% MEM NEAA (Gibco, 11140-050), 0.1% Beta-Mercaptoethanol (LifeTech, 21985-023), 10^6^ U/ml LIF (MiltenyiBiotec, 130-095-775) and 100 U/ml Penicillin-Streptomycin (Gibco, 15140122) at 37°C and 5% CO_2_.

To induce degron-mediated protein depletion, dTAG-13 (Tocris, 6605) and dTAG-V1 (Tocris, 6914) were dissolved in dimethyl sulfoxide (DMSO) and used as an equimolar mixture at a final concentration of 500 nM (dTAG-13) and 1000 nM (dTAG-V1) for the indicated periods of time.

For siRNA transfection, HEK293T cells were seeded in six-well dishes and transfected 24 h later at about 40% confluence using the DharmaFECT 4 Transfection Reagents (Mirus, T-2001-01) with a pool of small interfering RNAs (siRNAs) targeting RMP24 (Horizon, M-016886-01-0005) or a non-targeting siRNA pool (Horizon, D-001206-13-05) at 50 nM.

To monitor the rate of cell growth, cells were seeded at 5000 per well in a 48-well plate. At each indicated time point, cells were harvested by digestion with 0.25% trypsin-EDTA (Gibco, 2661762) from individual wells, and then counted using a hemocytometer (Fisher Scientific, 0267151B). Cell viability was determined via staining with Trypan Blue (Sigma, T8154-100ML).

To derive Rmp64- or RPP21-degradable cell lines, degron tag (FKBPF36V)-containing knock-in cassettes were introduced homozygously into the last coding exon of the *Rmp64* gene in mESC E14 cells or into the first coding exon of the RPP21 gene in HEK293T cells, as previously described [31]. Modified plasmids pCRIS-PITChv2-BSD-dTAG (Addgene, 91792), pCRIS-PITChv2-dTAG-BSD (Addgene, 91795), and pX333 (for simultaneous delivery of the PITCh and gene-targeting gRNAs; Addgene, 64073) were introduced into the cells via transfection.

The HEK293T Rmp64-degradable cell line was generated via infection with lentivirus generated from the pLEX_305-C-dTAG plasmid (Addgene, 91798) to drive degradation tag (dTAG)-inducible expression of Rmp64, and modified pX333 (for delivery of RMP64 gene-targeting gRNAs; Addgene, 64073) were introduced into the cells via transfection to knockout the endogenous *RMP64* gene.

HEK293T cells stably expressing Flag-HA-RPP25 and E14 mESCs stably expressing Flag-HA-Rpp25, Rpp14-3 × Flag, Rpp21-3 × Flag, Rmp24-3 × Flag, or Rmp64-3 × Flag were derived via infection with lentivirus made from modified plasmid pUltra-hot (Addgene, 24130) and sorting based on expression of the Cherry protein.

### Metabolic labeling and visualization of pre-rRNA transcription

#### Radioactive labeling

Metabolic labeling of pre-rRNA with ^32^P was performed as described previously [32], with minor modifications. Briefly, RMP64-degradable HEK293T cells were cultured in six-well plates and treated with either DMSO or dTAG for the indicated times. Cells treated with Actinomycin D (5 μM, 30 min) served as negative controls. Prior to labeling, cells were starved for 1 h in phosphate-free DMEM (Gibco, 11971025) supplemented with 10% dialyzed FBS (Gibco, A3382001). Labeling was performed by incubating cells for 1 h in the same medium containing 20 μCi/ml of ^32^P-orthophosphoric acid (Revvity, NEX053H001MC). Total RNA was extracted using TRIzol reagent (Invitrogen, 15596018) according to the manufacturer’s instructions. RNA samples were heated for 5 min at 70°C in 30 μl of 0.4 M formaldehyde/50% formamide/1 × TT buffer (30 mM tricine, 30 mM triethanolamine)/0.5 mM ethylenediaminetetraacetic acid (EDTA), then cooled to room temperature. Fifteen microliters of each sample was separated on 1% agarose/0.4 M formaldehyde/1 × TT gels (15 cm × 15 cm × ∼0.5 cm) in 1 × TT buffer, run at 130 V for 5 min followed by 100 V for 3.5 h. The gel was then dried using a Bio-Rad Model 583 gel dryer for 1 h. The dried gel was exposed to a storage phosphor screen (GE Healthcare, 0146-931) in an autoradiography cassette (Fisher Scientific, FBAC810) overnight at −20°C. Phosphor screens were imaged the following day using a Typhoon imager (Cytiva).

#### 5-Ethynyluridine labeling and immunofluorescence

For immunofluorescence analysis of newly transcribed RNA, RMP64-degradable HEK293T cells treated with either DMSO or dTAG were pulse-labeled with 1 mM 5-ethynyluridine (5-EU; Jena Bioscience, CLK-N002-10) for 30 min. After labeling, cells were fixed with 4% formaldehyde, permeabilized with saponin-based wash buffer [0.1% saponin, 0.1% bovine serum albumin (BSA), 0.01% NaN_3_ in phosphate buffered saline (PBS)], and incubated with a click-reaction cocktail (1 mM CuSO_4_, 43 mM Tris, 129 mM NaCl, 5.6 mM sodium ascorbate, and 50 μM CalFluor 488 azide; Vector Laboratories, 1369-1) for 30 min at room temperature in the dark. Cells were subsequently washed once with wash buffer (0.5 mM EDTA, 2 mM NaN_3_ in PBS) and once with PBS, blocked with 5% FBS in PBS, and incubated with primary antibodies overnight at 4°C. After washing, cells were incubated with fluorochrome-conjugated secondary antibodies for 1 h at room temperature, then mounted with VECTASHIELD Antifade Mounting Medium containing DAPI (Vector Laboratories, H-1200-10). Images were acquired using a Leica DMi8 microscope. Cells treated with Actinomycin D (5 μM, 30 min) served as negative controls.

### Sodium dodecyl sulfate–polyacrylamide gel electrophoresis and western blot analysis

Western blots were performed as described previously [32]. Briefly, whole cell lysates were run on 15% sodium dodecyl sulfate (SDS)–polyacrylamide gels and transferred to supported nitrocellulose membranes (Bio-Rad) at 145 V for 1.5 h. Membranes were then blocked for 1 h in 5% non-fat dry milk in tris-buffered saline with Tween-20 (TBST; 137 mM NaCl, 20 mM Tris, pH 7.6, 0.1% Tween-20), rinsed three times for 5 min with TBST, and incubated with primary antibodies (1:1000 in 3% BSA in TBST) overnight at 4°C. Blots were washed in TBST 5 min for three times, incubated with HRP-conjugated secondary antibodies in 5% milk in TBST for 1 h, and then washed again before developing. Beta-actin was detected by incubating blots with HRP-conjugated beta-actin antibody (1:15 000 in 5% milk in TBST) for 15 min, followed by washing and detection of the HRP signal with SuperSignal™ West Pico PLUS Chemiluminescent Substrate (Thermo Scientific, 34578).

### Immunofluorescence

Immunofluorescence was performed as previously described [32]. Briefly, cells were fixed in 4% formaldehyde in PBS for 10 min at room temperature, permeabilized in 0.5% Triton X-100 in PBS, blocked in 5% FBS in PBS, and probed with primary antibodies at 4°C overnight. The cells were then probed with fluorochrome-conjugated secondary antibodies for 1 h at room temperature and mounted using VECTASHIELD Antifade Mounting Medium with DAPI (Vector Laboratories, H-1200-10). Images were taken with the LEICA DMi8 microscope.

### Antibodies

Antibodies specific for NPM1 (Santa Cruz Biotechnology, sc-70392), RPP20 (Santa Cruz Biotechnology, sc-244043), RPP40 (Proteintech, 11736-1-AP), RPP21 (Proteintech, 16377-1-AP), RPP25 (Sigma, HPA046900-100UL), RMP24 (Sigma, HPA065505-100UL), HA tag (Cell Signaling Technology, 3724S), FLAG tag (Sigma, F1804-200UG), alpha Tubulin (Santa Cruz Biotechnology, sc-5286), and beta Actin (Sigma, A3854) were used in this study.

### Quantitative polymerase chain reaction (qPCR) analysis

qPCR was performed as previously described [32]. Briefly, total RNA was extracted from samples using TRIzol Reagent (Themo Fisher, 15596018) and Direct-zol RNA Miniprep (Zymo Research, R2050) according to the manufacturer’s instructions. Complementary DNA (cDNA) was prepared from equal amounts of RNA using PrimeScript RT Reagent Kit (Takara, RR037A) following manufacturer’s instructions. qPCR was performed using PowerUp SYBR Green Master Mix (Thermo Fisher, A25742) and oligonucleotide primers, listed in Supplementary Table S2, on the CFX Connect Real-Time PCR Detection System at the annealing temperature of 63°C. Relative mRNA levels were normalized to the expression of the human *ACTB* or mouse *Actb* genes in HEK293T and mESCs, respectively.

### Northern analysis

Northern blots with agarose/formaldehyde gels were performed as described previously [6]. All Northern probes, listed in Supplementary Table S2, were DNA oligonucleotides (Thermo Fisher) 5′ end-labeled with ^32^P gamma-ATP (PerkinElmer, NEG502A250UC) using the T4 polynucleotide kinase (New England Biolabs, M0201L) according to the manufacturer’s instructions. Briefly, 10–20 μg of total RNA was heated for 5 min at 70°C in 0.4 M formaldehyde/50% formamide/1 × TT (30 mM tricine, 30 mM triethanolamine)/0.5 mM EDTA, cooled to room temperature in a thermocycler, and separated on 1% agarose/0.4 M formaldehyde/1 × TT gels (15 cm × 15 cm × 0.5 cm) in 1 × TT at 130 V for 5 min followed by 100 V for 3.5 h. The gels were rinsed with deionized water, soaked while gently shaking for 15 min in 50 mM NaOH, rinsed again with deionized water, and while gently shaking in 6 × SSC for 15 min. RNA was capillary-transferred to Hybond N+ membranes (Cytiva, RPN203B) in 6× SSC overnight at room temperature. After transfer, membranes were cross-linked twice at 1200 mJ/cm^2^ before prehybridization in 10 ml of the hybridization buffer (Invitrogen, AM8670) for 60 min at 42°C. Probes (ITS1_3′ end, mITS1_3′ end) were then hybridized with the membrane overnight at 42°C. The next day, the membrane was washed sequentially with the wash buffer 1 (2× SSC, 0.1% SDS), wash buffer 2 (1× SSC, 0.1% SDS), and wash buffer 3 (0.1× SSC), once each for 30 min. Then membrane was developed using the storage phosphor screen (GE Healthcare, 0146-931) in an autoradiography cassette (Fisher Scientific, FBAC810) overnight at −20°C. The next day, the phosphor screen was imaged with a Typhoon imager (Cytiva).

### RNA fluorescence *in situ* hybridization

Fluorescence *in situ* hybridization (FISH) probes were ordered from Integrated DNA Technologies and are listed in Supplementary Table S2. The HEK293T Rmp64-degradable cells treated with DMSO or dTAG and WT HEK293T cells transfected with siCTRL or siRMP24 were probed with the RNA FISH probes labeled with cyanine dyes Cy5 or Cy3 according to the manufacturer’s instructions available online at www.biosearchtech.com/stellarisprotocols.

### Co-immunoprecipitation from cell lysates

The co-IP experiments were performed as previously described [32]. Briefly, E14 mESCs were harvested, washed once with PBS, and lysed in whole-cell lysis buffer (20 mM HEPES-KOH, pH 7.9, 10% glycerol, 300 mM KCl, 0.1% IGEPAL, 1 mM dithiothreitol (DTT)) supplemented with cOmplete Protease Inhibitor Cocktail (Millipore Sigma, 11697498001) for 30 min at 4 °C. Supernatants were cleared of debris by a 30-min centrifugation at 17 000 × *g* at 4°C. The lysates were then mixed with an equal volume of no-salt lysis buffer (20 mM HEPES-KOH, pH 7.9, 10% glycerol, 0.1% IGEPAL, 1 mM DTT) supplemented with cOmplete Protease Inhibitor Cocktail to lower the final salt concentration to 150 mM KCl (IP buffer), added to ANTI-FLAG® M2 Affinity Gel (Millipore Sigma, A2220), and rotated for 2 h at 4°C. After the IP, the beads were washed thoroughly with the IP buffer and the bound proteins were eluted with 150 μg/ml Flag (DYKDDDDK) peptide (GenScript, RP10586) in thermomixer at 4 °C, shaking at 1250 rpm for 1 h. The eluates were analyzed by western blotting.

### Protein complex purification

To purify protein complexes of Flag-HA-RPP25, Flag-HA-Rpp25, Rpp21-3 × Flag, Rmp64-3 × Flag, and Rmp24-3 × Flag, ∼10 million (for anti-Flag IP) or 300 million (for anti-Flag IP followed by anti-HA IP) HEK293T cells or E14 mESCs per experiment were harvested, flash-frozen in liquid nitrogen, and stored at −80°C until use. Cells were resuspended in buffer A (20 mM HEPES-KOH, pH 7.9, 10% glycerol, 300 mM KCl, 0.1% IGEPAL, 1 mM DTT, cOmplete Protease Inhibitor Cocktail; 100 μl of buffer A was used per 10^6^ cells) and rotated at 4°C for 30 min. The lysates were centrifuged at 17 000 × *g* for 30 min at 4 °C, supernatants were collected, and then 250 μl ANTI-FLAG® M2 Affinity Gel (Millipore Sigma, A2220) was added and the mixture was rotated for 2 h at 4°C. The affinity gel was then washed four times with buffer 1 (20 mM HEPES-KOH, pH 7.9, 10% glycerol, 300 mM KCl, 0.1% Tween-20, 1 mM DTT, cOmplete Protease Inhibitor Cocktail), followed by four washes with buffer 2 (20 mM HEPES-KOH, pH 7.9, 10% glycerol, 150 mM KCl, 0.1% Tween-20, 1 mM DTT, cOmplete Protease Inhibitor Cocktail). The bound proteins were eluted with Flag peptide (150 μg/ml; GenScript, RP10586) in thermomixer at 4°C, shaking at 1250 rpm for 1 h. The eluted proteins were analyzed by mass spectrometry. For tandem affinity purification, the Flag eluate was mixed with anti-HA magnetic beads (Thermo Fisher Scientific, 88837) and rotated for 2 h at 4°C. The beads were washed four times with buffer 2, and proteins were eluted using HA peptide (200 μg/ml; GenScript, RP11735) by shaking the beads in thermomixer at 1400 rpm for 45 min at 30°C. The eluted proteins were then analyzed by mass spectrometry.

### Immunoprecipitation mass spectrometry

Half of the elution sample was combined with 10 mM DTT and 5% SDS, then incubated at 95°C for 10 min to reduce and denature proteins. Following SP3 sample preparation [33], SP3 beads and 100% acetonitrile were added to the samples to precipitate proteins. SP3 beads were washed twice separately with pure acetonitrile and 70% ethanol to clean up the sample. They were resuspended with 40 μl 100 mM triethylammonium bicarbonate. The sample was digested overnight in 2 μl trypsin (200 ng/μl) at 37°C. The trypsin-digested sample was dried down to concentrate and resuspended in 4 μl 0.1 formic acid (FA) in H_2_O. For LC-MS/MS analysis, 1 μl of each sample was analyzed by the Exploris 480 mass spectrometer coupled with nLC Vanquish Neo UHPLC. Peptides were separated using a 25 cm × 75 μl column (Aurora Ultimate C18 1.7 μm). The flow rate was 200 nl/min on 70-min gradient (65 min active gradient). The Solent A was 0.1 FA in water, the solvent B was 80% of acetonitrile with 20% 0.1 FA in H_2_O. The gradient steps are as follows: (i) 14%–36% over 65 min for tryptic peptides separation; (ii) 95% B for 5 min to remove the residues from the column.

The MS/MS-based proteomics data were acquired on an Orbitrap Exploris 480 mass spectrometer operated in DDA mode. The ion source was operated in the positive ion mode with 1700 V spray voltage. The temperature of the ion transfer tube was 250°C. The full scan parameters were as follows: MS1 scans with 120 000 resolutions with a scan range of 350–1200 m/z; auto mode of maximum injection time; standard AGC target. Precursors were selected for fragmentation subject to intensity threshold of 5 × 10^3^, charge states between +2 and +5, and the exclusion duration was 45 s. The Data-Dependent MS2 scan was performed with a cycle time of 1 s. The MS2 scan was set to 0.7 m/z isolation window, 36% HCD collision energies, 45 000 resolution, auto mode of maximum injection time and 200% AGC target.

The raw data were analyzed by MaxQuant (v2.6.1.0). The mouse protein database was downloaded from UniProt (2025). The maximum missed cleavages were set up to 2 for tryptic peptides. Oxidation (M) and acetylation (Protein N-term) were added as variable modification. The maximum number of modifications per peptide was set up to 5. The 5% peptide FDR and 5% protein FDR were applied before summarizing peptides into proteins.

### RNA immunoprecipitation and qPCR

Briefly, WT, Rpp14-3 × Flag, Rpp21-3 × Flag, Rmp64-3 × Flag, and Rmp24-3 × Flag expressing mESCs were seeded on 10-cm plates, harvest at 80% density, washed twice with PBS, and pelleted by centrifugation at 1200 rpm for 5 min. Cell pellets were resuspended in 1000 μl of pre-chilled buffer A (20 mM HEPES-KOH pH 7.9, 10% glycerol, 300 mM KCl, 0.1% IGEPAL, 1 mM DTT, cOmplete Protease Inhibitor Cocktail) and rotated at 4°C for 30 min. The lysates were centrifuged at 17 000 × *g* for 30 min at 4°C, supernatants were collected, and aliquots of 50 μl were combined with 500 μl of TRI Reagent and stored at −80°C as input controls. The remaining lysate was mixed with 50 μl of ANTI-FLAG® M2 Affinity Gel (Millipore Sigma, A2220), and the mixture was rotated for 2 h at 4°C. The affinity gel was then washed four times with buffer 1 (20 mM HEPES-KOH, pH 7.9, 10% glycerol, 300 mM KCl, 0.1% Tween-20, 1 mM DTT, cOmplete Protease Inhibitor Cocktail), followed by four washes with buffer 2 (20 mM HEPES-KOH, pH 7.9, 10% glycerol, 150 mM KCl, 0.1% Tween-20, 1 mM DTT, cOmplete Protease Inhibitor Cocktail). The bound RNPs were eluted with Flag peptide (150 μg/ml; GenScript, RP10586) in thermomixer at 4°C, shaking at 1250 rpm for 1 h. For each sample, 50 μl of the Flag eluate was combined with 500 μl of TRIzol Reagent (Themo Fisher, 15596018) and stored at −80°C. RNA was isolated using Direct-zol RNA Miniprep kit (Zymo Research, R2050) according to the manufacturer’s instructions. cDNA was prepared from equal amounts of RNA using PrimeScript RT Reagent Kit (Takara, RR037A) following manufacturer’s instructions. qPCR was performed on the CFX Connect Real-Time PCR Detection System at the annealing temperature of 63°C using PowerUp SYBR Green Master Mix (Thermo Fisher, A25742) and the oligonucleotide primers listed in Supplementary Table S2. Relative RNA levels were normalized to the expression levels of the Actin gene. The relative enrichment of *Rpph1* and *Rmrp* in RNA purified from subunit-overexpressing cells was quantified by normalizing their levels to inputs and referencing them to the enrichment in non-overexpressing (WT) cells.

### AlphaFold prediction methods and Foldseek searches

Predictions of individual protein subunits of RNases P or MRP were generated with AlphaFold 2 [34] via the Google Colab notebook (https://colab.research.google.com/github/sokrypton/ColabFold/blob/main/AlphaFold2.ipynb) [35] using the default parameters but specifying to relax all predicted structures using Amber. Predictions of the human RNase MRP were generated with AlphaFold3 [36] (https://alphafoldserver.com/welcome) using the default settings. Ten structures were predicted after providing the full-length sequences of the RNases P or MRP subunits, and aligned with ChimeraX [37] to assess prediction convergence. ChimeraX was also used to prepare all structural figures.

We used the Foldseek web interface (https://search.foldseek.com/search) [38], using as queries the experimentally determined structures of *Saccharomyces cerevisiae* Rmp1 and Snm1 proteins (PDB: 7C7A) [7].

### Individual-nucleotide resolution UV-crosslinking and immunoprecipitation

All iCLIP experiments were performed in replicates following the iCLIP2 protocol [39]. Briefly, E14 mESCs stably expressing Flag-tagged Rmp64, Rpp14, Pop5, or Rpp21 were grown in 10-cm plates and harvested at 85% confluence. The cells were washed with ice-cold PBS and irradiated with UV light at 254 nm on ice. The irradiated cells were scraped, aliquoted into three 2 ml tubes, and centrifuged at 5000 × *g* for 2 min at 4°C. The supernatant was removed, and the cell pellets were flash-frozen in liquid nitrogen and stored at −80°C until use. Immunoprecipitation of the crosslinked RNP complexes was carried out using anti-Flag antibody (Millipore Sigma, F1804). The complete iCLIP experiment, including deep sequencing of the prepared cDNA libraries, was repeated in four replicates for each subunit.

### iCLIP data processing

The iCLIP data were processed essentially as previously described [40]. FastQC (v0.11.9, https://www.bioinformatics.babraham.ac.uk/projects/fastqc/) was used to assess the data quality and high quality data were provided with FASTX Toolkit (v0.0.13, http://hannonlab.cshl.edu/fastx_toolkit/. parameters: -Q 33 -q 10 -p 100). Flexbar (v3.5.0) [41, 42] was utilized to demultiplex data based on their indexes, followed by mapping to the mm10 genome with STAR genome aligner v2.7.3a [43]. Umi_tools v1.0.1 [44] was used to remove PCR duplicates. High-confidence crosslink sites were identified with PureCLIP v1.3.1 (parameter: -ld -nt 8) [45]. The crosslink sites identified in at least two replicates were kept and expanded to a ∼9-nt region as described previously [40]. Binding sites were annotated using GENCODE (vM25) comprehensive gene annotation. Bigwig files were produced with bedtools v2.31.1 [46] by shifting the reads 1 nucleotide upstream to identify the genuine crosslink sites and by normalizing to the whole library with RPM (reads per million). The IGV version 2.9.4 [47, 48] traces shown in the figures were summaries of all four replicates. RNase MRP specific peaks were those identified in Rmp64, Rpp14, and Pop5, but not in Rpp21 iCLIP datasets, whereas RNase P specific peaks were those identified in Rpp21, Rpp14, and Pop5, but not Rmp64 iCLIP datasets.

### Gene nomenclature

In coordination with the HUGO Gene Nomenclature Committee (HGNC), we proposed and received approval for the official naming of NEPRO as *RMP64* (HGNC:56 432) and C18orf21 as *RMP24* (HGNC:56 433). These new symbols reflect their specific association with RNase MRP (e.g. *RMP24* for ribonuclease MRP subunit p24) and follow established conventions for RNase P/MRP subunits. During this consultation, we also proposed renaming the only known RNase P-specific subunit *RPP21*, and HGNC approved changing its name from “ribonuclease P/MRP subunit p21” to “ribonuclease P subunit p21.”

The RPP root symbol itself was introduced in the late 1990s to name several RNase P subunits lacking established yeast orthologs. Notably, Eder *et al.* [49] and Jarrous *et al.* [50] first used Rpp-based nomenclature in human to describe what are now known as *RPP20* (alias of *POP7*), *RPP30*, *RPP38*, and *RPP40*. They also noted *Rpp14* and *Rpp25* as encoding putative RNase P subunits, later confirmed by additional studies [51, 52]. The HGNC has since adopted the RPP root symbol for six subunits: *RPP14*,*RPP21*,*RPP25*,*RPP30*,*RPP38*, and *RPP40*. The remaining four human subunits—*POP1*,*POP4*,*POP5*, and *POP7*—were named based on their clear orthology to yeast *POP* genes (“processing of precursor RNAs”).

When it became evident that RNase P and RNase MRP share multiple subunits [53], HGNC updated the gene names (but not symbols) of shared subunits to reflect this (e.g. *RPP14*, previously “ribonuclease P subunit p14,” became “ribonuclease P/MRP subunit p14”). By contrast, the *RMP* root symbol is reserved for genes encoding subunits specific to RNase MRP. While newly adopted for human genes, the *RMP* symbol is not novel: the yeast RNase MRP-specific subunit Rmp1 was named in *S. cerevisiae* in 2005 [26], providing both precedent and evolutionary continuity.

The HGNC maintains curated gene group pages listing the subunits of the nuclear RNase P (https://genenames.org/data/genegroup/#!/group/3374), RNase MRP (https://genenames.org/data/genegroup/#!/group/3375) complexes.

We also note that two other groups reported the discovery of *RMP24* and *RMP64* at approximately the same time [54, 55]. In addition, the group of Ming Lei independently identified the same subunits through integrative structural studies of human RNase MRP. To minimize confusion and align the field, we coordinated the naming of these genes through HGNC.

## Results

### Structural homology identifies two novel candidate subunits of metazoan RNase MRP

Recent cryo-EM studies in *S. cerevisiae* have revealed a considerable overall architectural similarity between RNase P and RNase MRP RNPs, as well as the preserved catalytic RNA core inherited from their common ancestor (Fig. 1B) [7, 18]. In addition, these analyses revealed incorporation of new RNA elements as well as two protein subunits, Rmp1 and Snm1, that contributed to the establishment of yeast RNase MRP as an enzyme with substrate specificity distinct from RNase P. It is not known whether putative orthologs of the yeast RNase MRP-specific proteins might be components of metazoan RNase MRP.

As sequence-based searches for human orthologs of Rmp1 or Snm1 found no obvious candidates, we asked whether such proteins might exist based on their structural homology. We used Foldseek to query the cryo-EM structures of the two yeast proteins within RNase MRP against thousands of experimentally determined or computationally predicted protein structures [38]. This search identified the N-terminal helical bundle of the little studied nucleolar protein NEPRO [56–58] as distinctly the closest structural match to the yeast Rmp1 in all inspected metazoans species, despite the limited amino acid sequence conservation (Fig. 1C and Supplementary Fig. S1A and B). A similar search of the yeast Snm1 protein revealed the most extensive homology with two human proteins: the RNase P-specific subunit RPP21 and an uncharacterized protein C18ORF21 (Fig. 1D). Interestingly, in yeast, the RNase MRP-specific Snm1 is structurally most homologous with the RNase P-specific Rpr2 (Supplementary Fig. S1C), and both proteins are similarly nestled in their RNP complexes between Pop4 and Pop3 (Fig. 1B) [7, 18]. It is thus plausible that RPP21 and its homolog C18ORF21 occupy the same relative positions in metazoan RNase P and RNase MRP, respectively (Supplementary Fig. S1D–F). We proposed the gene symbols RMP64 (for NEPRO) and RMP24 (for C18ORF21) to the HGNC, which formally approved these designations. This nomenclature is used throughout the remainder of the manuscript (see “Materials and methods” section).

We used AlphaFold to generate structure predictions of human RMP64 and RMP24 in complex with the previously annotated subunits of the human RNase MRP (Supplementary Fig. S2A) [3]. These predictions suggested that twelve protein subunits, including RMP64 and RMP24, sequentially contact one another and tightly wrap around *RMRP*, much like the orthologous proteins of the yeast RNase MRP interlink together to stabilize *Nme1*, the yeast RNase MRP catalytic RNA (Fig. 1B and Supplementary Fig. S2A) [7, 18]. However, unlike the hook-shaped architecture of RNase MRP in yeast, the predictions of human RNase MRP uniformly indicate a more closed, rigid conformation of the RNP that is stabilized by extensive interactions of RMP64 and RMP24 with *RMRP* and with one another (Supplementary Fig. S2A–C). Additionally, all predictions show that the structures of the catalytic (C) domain in the human *RMRP* and yeast *Nme1* are nearly identical, while suggesting entirely different topologies of the specificity (S) domain between the two species (Supplementary Fig. S2D) [3, 7, 18, 59].

A comparison of the cryo-EM structure of the *S. cerevisiae* RNase MRP holoenzyme with structural predictions of its human counterpart points to striking similarities in the way that both RNase MRP-specific subunits are integrated into the enzyme. In both human and yeast, the extended N-terminal domain of RPP29/Pop4 wraps around one side of the helical bundle of RMP64/Rmp1, while POP1/Pop1 contacts the bundle from the opposite side (Fig. 1E and Supplementary Fig. S2A). All generated predictions further suggest intimate interactions of RMP64 with *RMRP* and position RMP64 atop the catalytic RNA pseudoknot (Fig. 1E and Supplementary Fig. S2C). This resembles the placement of Rmp1 in yeast RNase MRP, where it forms part of the substrate-binding groove and facilitates recognition of single-stranded RNA substrates (Supplementary Fig. S2B) [7, 18]. At the other end of the enzyme, Snm1 interacts with Pop4 and Pop3 in yeast in a similar fashion as RMP24 is predicted to interact with RPP29 and RPP38 in humans (Fig. 1B and F, and Supplementary Fig. S2A). However, compared to the Snm1 interaction with the yeast RNase MRP RNA, the predictions suggest considerably more extensive contacts between RMP24 and the human RNase MRP RNA (Fig. 1F and Supplementary Fig. S2B and C).

Together, these analyses identify two novel candidate subunits of the metazoan RNase MRP and predict their RNP-specific structural roles.

### RMP64 and RMP24 are constitutive components of RNase MRP but not RNase P

**Figure 2.**
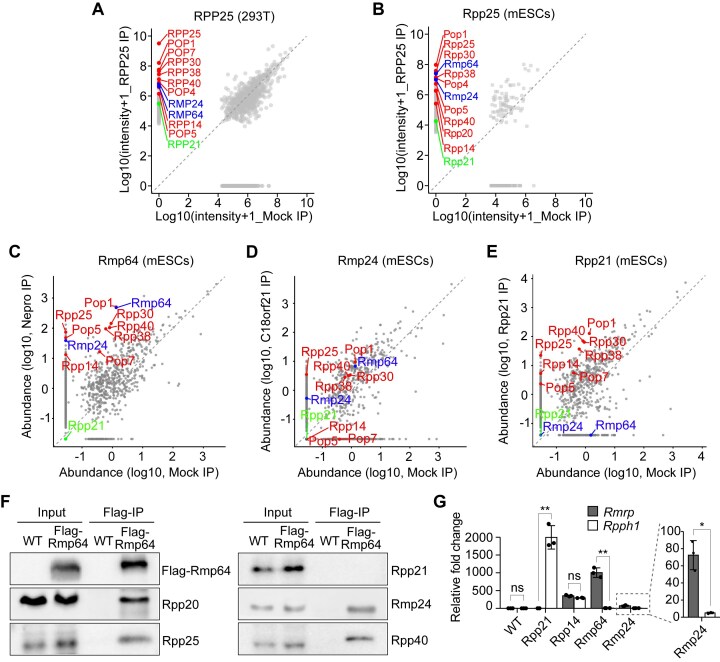
RMP64 and RMP24 are constitutive components of RNase MRP but not RNase P. Results of mass spectrometry analyses comparing summarized intensities for each protein detected in tandem affinity-purified protein complex (anti-Flag IP followed by anti-HA IP) of RPP25 and mock control in HEK293T cells (**A**) and mouse embryonic stem cells (mESCs) (**B**). RNase MRP protein subunits in common to RNase P are highlighted in red, the RNase P-specific RPP21 is in green, and the two newly identified RNase MRP-specific subunits, RMP64 and RMP24, are in blue. Results of mass spectrometry analyses comparing abundances of each protein detected in affinity-purified protein complex (anti-Flag IP) of RMP64 (**C**), RMP24 (**D**), or Rpp21 (**E**) and mock control in mESCs. Protein subunits of RNases P and MRP were highlighted as in panels (A) and (B). (**F**) Co-IP of endogenous RNase P and/or MRP protein subunits with Rmp64 from lysates of mESCs stably expressing 3×Flag-Rmp64. Precipitated proteins were detected by western blot analysis. (**G**) RNA immunoprecipitation (RIP) analysis of *Rmrp* and *Rpph1* co-precipitated with Flag-tagged Rpp21, Rpp14, Rmp64, or Rmp24 from lysates of mESCs. Relative fold change of *Rmrp* or *Rpph1* in each RIP experiment compared to wild-type (WT) control was determined using quantitative polymerase chain reaction (qPCR; *n* = 3). Data are shown as mean ± SD. *, *P* < .01; **, *P* < .001 (two-tailed Student’s *t* test); ns, not significant.

To confirm the predicted subunit status of RMP64 and RMP24 in RNase MRP, we sought to validate the association of each protein with the known subunits of the enzyme in human and mouse cells. We used affinity-purification mass spectrometry (AP-MS) to enable enrichment and detection of proteins that stably associate with different ectopically expressed tagged RNase P and/or RNase MRP subunits, which we used as baits. Tandem AP-MS analysis of the complex formed by the common subunit RPP25 in HEK293T or mESCs found among the most highly and specifically enriched proteins all subunits annotated as common to both enzymes, the RNase P-specific RPP21, as well as RMP64 and RMP24 (Fig. 2A and B). These proteins were also overrepresented in other complexes that we purified from mESCs, except that Rpp21 was not detected in the Rmp64- or Rmp24-nucleated complexes and, vice versa, Rmp64 or Rmp24 were not detected in the Rpp21 complex (Fig. 2C–E). We used co-IP/western analysis to validate the coexistence of Rmp64 and Rmp24, but the absence of Rpp21 within the same protein complex (Fig. 2F).

Given that RNases P and MRP use distinct catalytic RNA molecules, *RPPH1* and *RMRP*, respectively, we reasoned that any RNase MRP-specific protein subunit should preferentially associate with the latter. Markedly, RIP analyses of Rmp64 or Rmp24 in mESCs revealed an overwhelming binding preference for *Rmrp* compared to *Rpph1* for both proteins, whereas Rpp21 showed the expected specificity for *Rpph1* (Fig. 2G). Thus, the strong associations of both Rmp64 and Rmp24 with all known RNase MRP subunits, as well as with one another and with the RNase MRP RNA, support our structural analyses and identify Rmp64 and Rmp24 as specific protein subunits of metazoan RNase MRP.

### The *in vivo* RNA binding specificity of RNase MRP

We asked whether the specific association of the newly identified subunits with RNase MRP but not RNase P could be leveraged to shed light on the RNA-targeting specificity of RNase MRP in mammalian cells. We considered that in yeast, a short single-stranded segment of the pre-rRNA’s ITS1 region—a known substrate of the yeast RNase MRP—is accommodated deep within the catalytic center of the enzyme via direct contacts with several protein subunits, in addition to interactions with the *Nme1* RNA (Fig. 1A and Supplementary Fig. S2B) [7]. In fact, an earlier study of the structural organization of yeast RNase MRP demonstrated that many interactions between its protein subunits and substrate RNA could be successfully captured *in vitro* using UV light-mediated crosslinking [22]. To detect potential protein-RNA substrate interactions of mammalian RNase MRP *in vivo*, we carried out individual nucleotide resolution UV crosslinking and immunoprecipitation (iCLIP) [39, 60] of tagged RNase P and/or MRP subunits in mESCs.

**Figure 3.**
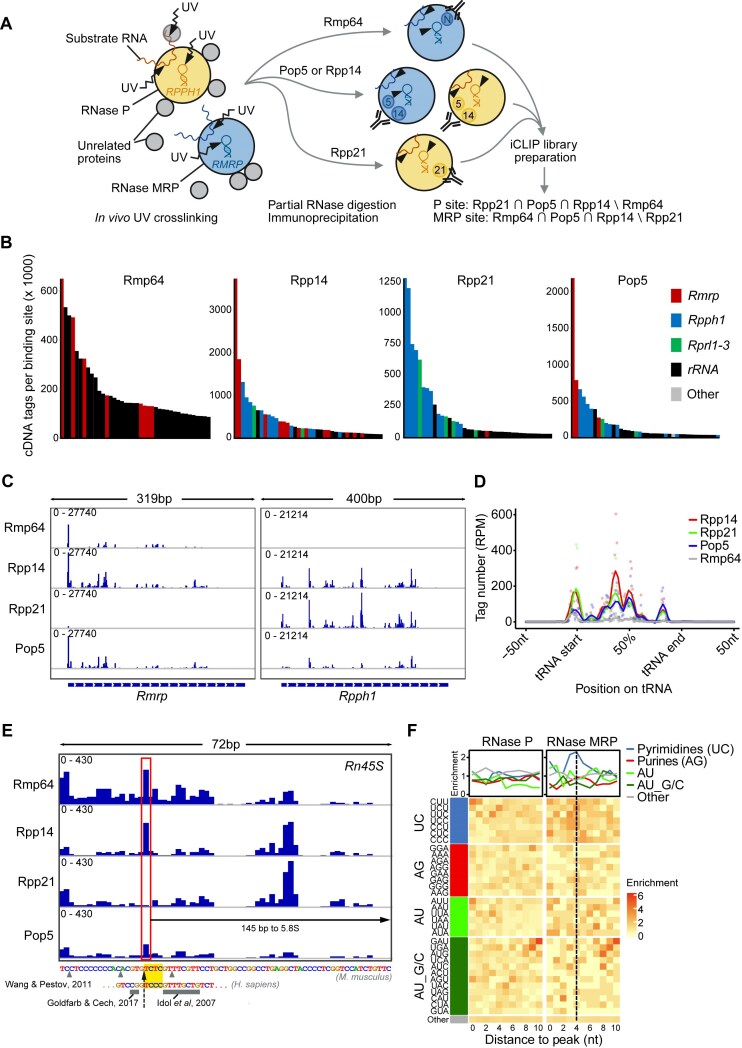
The *in vivo* RNA binding specificity of RNases P and MRP. (**A**) Schematic representation of the iCLIP strategy to determine RNA-binding specificities of RNases P and MRP. (**B**) Top RNA binding sites in each iCLIP dataset. The 40 binding sites with the highest number of unique cDNA tags (per binding site) are shown, ordered by tag count from left to right. Gencode vM25 annotations of individual binding sites are shown. (**C**) Crosslinking profiles on *Rmrp* and *Rpph1* shown for each iCLIP dataset. Numbers in each trace are counts per million values. (**D**) Proportional metatranscript analysis of iCLIP data showing the positional frequency of crosslink events on tRNAs. Data points represent normalized crosslink events summarized over every percent of the tRNA length. RPM, reads per million. (**E**) Crosslinking profile in the ITS1 segment of the mouse Rn45S transcript (NR_046233.2) highlighting the sole RNase MRP-specific site (red rectangle) and its distance from the 5.8S rRNA. Previously mapped RNase MRP cleavage sites are indicated separately on the mouse transcript (gray arrowheads) [68] and the orthologous section of the human transcript (gray rectangles) [6, 67]. The yellow rectangle and the black dashed arrow indicate putative RNase MRP consensus recognition sequence and cleavage site, respectively. (**F**) Heatmaps illustrating positional frequencies of the indicated sets of trimers 10 nts downstream of the RNase P (left) and RNase MRP (right) binding site maxima. Plots above the heatmaps profile the mean enrichment of the different sets of trimers.

We achieved co-purification of most RNP protein subunits crosslinked to RNA by using stringent but nondenaturing conditions, which were previously shown to preserve relatively stable snRNP complexes (Supplementary Fig. S3A) [61]. High concentration of salt and detergents minimized co-purification of weakly associated proteins but preserved the more stable inter-subunit interactions of RNases P or MRP, as evident from multiple radioactive bands whose pattern was similar between iCLIP experiments (Fig. 3A and Supplementary Fig. S3A) [39, 61]. We prepared cDNA libraries from a broad distribution of crosslinked RNP complexes to maximize the diversity of recovered protein-RNA interactions (Supplementary Fig. S3A).

Data analysis revealed several thousand protein–RNA interactions, the majority of which were identified with high reproducibility in each iCLIP dataset (Supplementary Fig. S3B). Binding sites with the largest numbers of unique crosslink events were uniformly located on the catalytic RNAs of RNases P and/or MRP, with crosslinks from Rmp64 iCLIP mapping selectively to *Rmrp* and those from Rpp21 iCLIP mapping to *Rpph1*, whereas the analysis of Rpp14 or Pop5 showed strong binding to both catalytic RNAs (Fig. 3B). A further inspection of the crosslinking to *Rmrp* or *Rpph1* revealed complex but very similar RNA binding patterns across the different iCLIP datasets, consistent with the capture of RNP-wide and not merely subunit-specific protein-RNA interactions (Fig. 3C and Supplementary Fig. S3A). For a crosslinking peak to be ascribed to either RNase P or RNase MRP, we required that it be called in (i) exactly three out of four independent iCLIP datasets and (ii) one but not both enzyme-specific subunit datasets, i.e. Rpp21 or Rmp64 but not both (Fig. 3A). This requirement secured validity of binding sites by disregarding those that were either non-specific (e.g. called in each iCLIP dataset due to adventitious binding of a highly expressed transcript) or were specific to the subunit but not the RNP complex (e.g. Rmp64 possibly occurs in cells outside of RNase MRP and may bind RNA independently) [56].

In addition to binding of the catalytic RNA, we detected interactions of RNases P and MRP with several other RNA species, including rRNA, tRNA, and mRNA, as well as with different types of short and long non-coding RNA (Fig. 3B, Supplementary Fig. S3C and D, and Supplementary Table S1). To assess the extent to which these interactions may indicate recognition of specific RNA substrates by RNases P and MRP, we first examined the binding of each enzyme to their known RNA targets. We found that Rpp14, Rpp21, and Pop5, but not Rmp64, iCLIP cDNAs mapped extensively to multiple positions along the length of tRNAs, in line with pre-tRNA being a dominant substrate of RNase P but not RNase MRP (Fig. 3D, Supplementary Fig. S3E, and Supplementary Table S1) [3, 5, 62]. This included a prominent crosslinking peak just upstream of the annotated mature tRNA segments, which agrees with structural studies of RNase P in different organisms that show or predict critical protein interactions with the 5′ leader of the pre-tRNA substrates at the catalytic center [20, 21]. We also examined interactions with another documented RNA substrate of RNase P, *Malat1*, whose conserved triple helical 3′ end is produced by recognition and cleavage of a tRNA-like structure by RNase P [63, 64]. Strikingly, we found a dominant RNase P-specific binding site on the ∼7-kb *Malat1* transcript precisely at the annotated RNase P cleavage site within a short A-rich tract (Supplementary Fig. S3F).

While RNase P recognizes its substrates primarily based on their shape and by acting as a molecular ruler [20, 21, 65], little is still known about substrate recognition by RNase MRP, aside from its preference for a loosely defined consensus sequence in yeast, 5′-*RCRC-3′ (where * is the cleavage site and R is a non-obligate purine), in which a cytosine at position +4 is particularly important [7, 23]. To gauge whether iCLIP might, as in the case of RNase P, identify RNase MRP substrates based on their crosslinking pattern, we inspected pre-rRNA as the only *bona fide* RNase MRP substrate in mammalian cells (Fig. 1A) [6]. Despite high background signal, we identified a single RNase MRP-specific crosslinking site on the 13-kb pre-rRNA transcript based on the peak presence in Rmp64, Rpp14, and Pop5 but not Rpp21 iCLIP datasets (Fig. 3E). Markedly, this site was located in the 1-kb ITS1 segment, 145 nts upstream of 5.8S rRNA, in the vicinity of previous mappings of the RNase MRP cleavage site in the mouse and orthologous sequences in the human pre-rRNA (Figs. 1A and 3E) [1, 6, 66–68].

We also note that the RNA sequence immediately downstream of the crosslinking site in ITS1 may fulfill the reported substrate recognition requirement of yeast RNase MRP, containing cytosines at positions +2 and +4 relative to the peak at position +1 (Fig. 3E) [7, 23]. In fact, a consideration of all identified RNase P- or RNase MRP-specific binding sites indicated that the latter were distinctly enriched in U/C-containing motifs just downstream of the binding peak, showing maximum enrichment at position +4, whereas RNase P-specific peaks showed no such trend (Fig. 3F). This analysis suggests that iCLIP may be, as in the case of known RNA substrates (Fig. 3D and E, and Supplementary Fig. S3E and F), capturing crosslinks right at the sites of cleavage in the candidate RNA substrates (Fig. 3F). Future studies are warranted to investigate potential RNase P- or MRP-dependent cleavage of the identified target RNAs, along with the biological implications of such cleavage events.

### RMP64 and RMP24 are required for pre-rRNA processing by mammalian RNase MRP

**Figure 4.**
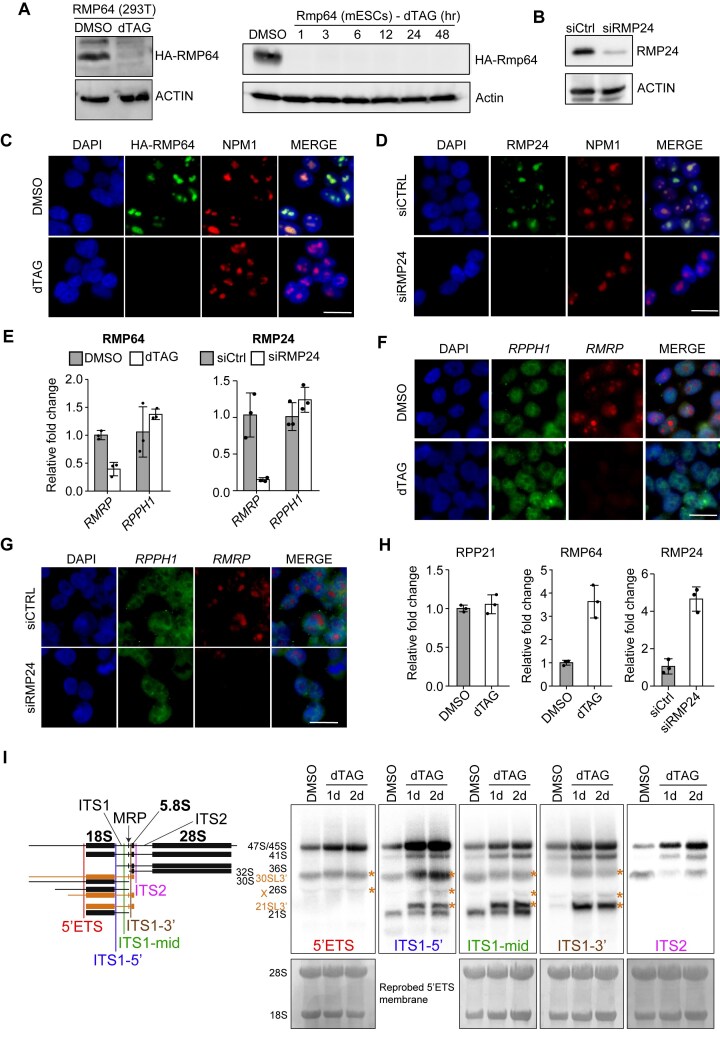
Mammalian RNase MRP requires RMP64 and RMP24 for pre-rRNA processing. (**A**) Depletion of HA-tagged RMP64 in HEK293T cells (left) or mESCs (right) engineered for rapid depletion of the protein subunit using the dTAG system [31, 69]. HEK293T cells were treated with dTAG for 48 h and mESCs as indicated prior to analysis by immunoblotting. Actin serves as a loading control (*n* = 3). (**B**) Depletion of RMP24 in HEK293T cells using siRNA at 2 days post transfection (*n* = 3). (**C**) The engineered HEK293T cells from panel (A) were analyzed by immunofluorescence using anti-HA antibody after treatment with DMSO or dTAG for 2 days. Antibodies targeting endogenous nucleophosmin (NPM1) and DAPI were used to visualize nucleoli and nuclei, respectively. (**D**) As in panel (C), only that RMP24 was depleted in WT HEK293T cells with siRNA for 2 days prior to the analysis. (E–G) Depletion of RMP64, as in panel (C), or RMP24, as in panel (D), reduces steady-state levels of *RMRP* but not *RPPH1*. Cells were analyzed by qPCR (**E**) or by fluorescence *in situ* hybridization (FISH) (**F**,**G**) (*n* = 3). Data in panel (E) are shown as mean ± SD. (**H**) qPCR analysis of the RNase MRP-targeted site in pre-rRNA in cells lacking RPP21, RMP64, or RMP24 (*n* = 3). Data are shown as mean ± SD. (**I**) Northern blot analysis of pre-rRNA processing intermediates in human cells upon acute RMP64 depletion. HEK293T cells engineered for dTAG-inducible degradation of RMP64 were treated with DMSO or dTAG for 1 or 2 days. Total RNA was extracted and analyzed by northern blotting using probes targeting the 5′ external transcribed spacer (5′ETS), 5′ end of ITS1, middle of ITS1, 3′ end of ITS1, and ITS2, as diagrammed in the schematic (left). Black bars represent canonical pre-rRNA intermediates; orange bars and asterisks denote noncanonical or alternatively processed species, based on prior studies [6, 72, 77]. Membranes stained with methylene blue (focusing on 18S and 28S rRNAs) are shown below each panel as a loading control. All RNA samples had RNA integrity numbers (RINs) of 9.3 or higher, as assessed by Agilent Bioanalyzer. Scale bars in (C), (D), (F), and (G) are 25 μm.

To understand the contribution of RMP64 and RMP24 to the enzymatic activity of mammalian RNase MRP, we evaluated the effects of their depletion in human or mouse cells. We rapidly and nearly completely removed RMP64 via targeted protein degradation using a conditional dTAG approach, while we most efficiently depleted RMP24 by siRNA-mediated gene silencing (Fig. 4A and B) [31, 69]. Both proteins distinctly localized to nucleoli, as would be expected from subunits of the overwhelmingly nucleolar RNase MRP [70, 71], though neither exerted any overt effect on nucleolar size or number upon depletion in HEK293T cells (Fig. 4C and D). Moreover, depletion of RMP64 or RMP24 led to a substantial decrease in the level of *RMRP* but not *RPPH1* (Fig. 4E–G), consistent with direct, stabilizing interactions that each of the two proteins are predicted to form with *RMRP* within the RNase MRP RNP complex (Fig. 1E and F, and Supplementary Fig. S2C).

We asked whether the processing of the key RNase MRP substrate, pre-rRNA, was also affected. A qPCR analysis of nuclear RNA using primers that amplify a region spanning the RNase MRP cleavage site in ITS1 found little difference upon depletion of the RNase P-specific RPP21 protein, but indicated severely compromised cleavage when either RMP64 or RMP24 were depleted (Fig. 4H and Supplementary Fig. S4A). Northern blot analyses confirmed a block in early pre-rRNA processing upon depletion of RMP64 in HEK293T and mES cells, showing accumulation of 47S/45S rRNA and other rRNA precursors consistent with a defect in cleavage of the ITS1 segment by RNase MRP (Fig. 4I and Supplementary Fig. S4B) [6, 72]. Accumulation of ITS1-retaining pre-rRNA, driven by its continued transcription at a reduced rate (Supplementary Fig. S4C and D), was also observed by fluorescence *in situ* hybridization analysis, which revealed a substantially increased ITS1 signal in nucleoli of RMP64-depleted cells (Supplementary Fig. S4E).

Finally, we found that the rapid depletion of RMP64 or RMP24 caused a proliferative arrest but preserved cellular viability for several days until termination of the experiment (Supplementary Fig. S4F and data not shown). The observed enduring cell viability agrees with previous studies demonstrating long-term survival of cells arrested by inhibition of rRNA biogenesis, including via inhibition of rRNA transcription and downstream processing [73–77]. Taken together, our structural analyses (Fig. 1), studies of protein-protein and protein-RNA interactions (Figs. 2 and 3), and *in vivo* functional studies (Fig. 4) elucidate the composition and function of RNase MRP in metazoans, paving the way to a better understanding of this essential RNA-based regulator of RNA processing.

## Discussion

Despite its essential role in rRNA processing, ancient evolutionary origin, and association with a series of severely debilitating diseases, metazoan RNase MRP remains an enzyme of unclear composition and substrate specificity. Our discovery of two human RNase MRP-specific protein subunits, RMP64 and RMP24, which are conserved across metazoans, provides a critical molecular handle for structural and functional characterization of RNase MRP. This includes a solution to the long-standing problem of distinguishing the protein composition of RNase MRP from its structurally and evolutionarily closely related RNase P. To carry out these studies, we generated cell lines for rapid, on-demand depletion of RNase P or MRP, as well as a database of candidate RNA substrates with annotated crosslinking sites of each enzyme.

We identify both human RNase MRP subunits, RMP64 and RMP24, as the closest predicted structural homologs of yeast Rmp1 and Snm1, respectively, despite poor sequence conservation. In fact, the similarity between the orthologous subunits is limited to the structural elements that anchor each yeast subunit into the RNase MRP complex: the N-terminal four-barrel bundle in RMP64/Rmp1 and the N-terminal helices and beta-sheets in RMP24/Snm1 (Fig. 1C and D). Notably, the same structural elements are consistently predicted to anchor RMP64 and RMP24 into the human RNase MRP complex at comparable relative positions as in the yeast complex and foster many similar interactions with the neighboring subunits and the catalytic RNA (Fig. 1E and F, and Supplementary Fig. S2B and C). However, RMP64, which is more than twice the size of Rmp1 in yeast, and RMP24 are predicted to form much more extensive contacts with the catalytic RNA than their yeast counterparts (Fig. 1F and Supplementary Fig. S2B and C) and directly interact with each other to ‘close’ the general hook-shaped architecture of the enzyme (Fig. 1B and Supplementary Fig. S2A) [7, 18]. We predict that these metazoan-specific protein–RNA interactions contribute to stabilization of the catalytic RNA, in line with the effects of RMP64 or RMP24 depletion in mammalian cells (Fig. 4). The evolution of RNase MRP from its last universal common ancestor with RNase P thus appears to have continued the trajectory of becoming progressively larger and more proteinaceous in eukaryotes (Fig. 1B and Supplementary Fig. S2A) [7, 78].

One pressing question regarding the biological functions of RNase MRP revolves around its RNA targeting repertoire. Despite the identification of two mRNA substrates in yeast [28, 29, 79], the quest to understand the RNA binding specificity of RNase MRP in higher organisms has been complicated by lack of knowledge of the enzyme's composition and its sharing of the known protein subunits with RNase P [3]. We circumvented these issues by pairing our identification of RNase MRP-specific protein subunits with iCLIP to allow for transcriptome-wide discovery of RNase P or RNase MRP-specific RNA binding sites (Fig. 3A) [39, 60]. Strikingly, this analysis not only captured the known RNA substrates of RNase P (tRNAs and *Malat1*) and RNase MRP (pre-rRNA), but in every case also pointed to a prominent crosslinking peak precisely at the expected site of cleavage (Fig. 3D, E and Supplementary Fig. S3F). This result indicates particularly efficient *in vivo* crosslinking of protein subunits to the RNA substrate at the catalytic core of each enzyme, consistent with results of the *in vitro* crosslinking experiments with yeast RNase MRP [22]. Generalized to all our identified RNase MRP target sites, such crosslinking preference would point to a pyrimidine-rich consensus sequence for mammalian RNase MRP (Fig. 3E and F), hypothetically 5′-*YCYC-3′ (where * is a potential cleavage site and Y is a non-obligate pyrimidine), contrasting with the 5′-*RCRC-3′ consensus determined for the yeast RNase MRP [7, 22].

We identified *Rmrp* and *Rpph1* as the most highly bound RNA molecules with over a million unique iCLIP cDNAs mapped at some of the strongest bound sites (Fig. 3B). Although such read depth may seem excessive, it was required to identify many of the weaker crosslinked RNA targets with confidence. Curiously, some of the strongest crosslinking peaks were located on transcripts of three RNase P RNA-like genes, *Rprl1-3*, which are homologous to but shorter than *Rpph1* (Fig. 3B) [80, 81]. Although these transcripts have not been functionally characterized, they are expressed in a highly tissue-specific manner and are speculated to represent, like RNase MRP, a case of functional diversification of RNase P [80, 81]. Our evidence for *in vivo* association of *Rprl1-3* transcripts with multiple subunits of RNase P but not the RNase MRP-specific Rmp64 suggests functional potential of the formed RNPs. This is noteworthy since homologs of *RPPH1* as well as *RMRP* have been found in other higher vertebrates, including humans [80, 81].

Our study finds essentially all canonical as well as numerous novel candidate RNA substrates for each endonuclease, totaling to 80 for RNase MRP and 311 (including 124 tRNAs) for RNase P, and spanning a diversity of different RNA biotypes (Supplementary Fig. S3C and Supplementary Table S1). The identified target RNAs along with their putative cleavage sites provide a valuable resource for much further research into the enigmatic *in vivo* functions of these essential RNPs. Our findings suggest that both RNase MRP and RNase P have been exapted for a far wider variety of additional roles in mammalian cells than previously appreciated. We speculate that these newly acquired functions contribute to the pressure to preserve these enzymes and continue their evolution. It is also conceivable that the human pathologies associated with mutations in RNase MRP RNA and RMP64 are influenced by impairment of such functions [9, 10, 57, 82, 83].

While our data do not directly address the evolutionary rationale for the structural complexity of eukaryotic RNase P and RNase MRP, this topic has been discussed in several reviews [3, 4, 8, 84]. Notably, constructive neutral evolution has been proposed as a likely mechanism by which RNase P and RNase MRP acquired their elaborate structures—through the accumulation of neutral mutations and interactions that increased molecular complexity without immediate selective benefit [84–87]. Such features may have persisted over evolutionary time and, in the case of these enzymes, could have been retained, co-opted, or further elaborated to support additional cellular functions. In particular, the expanded protein complement is thought to stabilize the catalytic RNA structure, enable binding to larger and more diverse RNA substrates, facilitate regulated interactions with other cellular machineries, and allow for compositional flexibility of the complexes [4, 10, 81, 84, 88, 89]. Our identification of numerous novel RNA targets—candidate substrates—of mammalian RNase MRP and RNase P lends credence to the idea that substantial substrate versatility and spatiotemporal regulation (e.g. relocalization of RNase MRP outside the nucleolus) are required for their functions. We further hypothesize that the RNase MRP-specific protein subunits RMP24 and RMP64 contribute to these regulatory and functional adaptations, helping to diversify RNase MRP’s activity relative to RNase P. Our characterization of the RNase MRP-specific subunits, together with the defined RNA targeting repertoires and cell lines for inducible depletion of each enzyme, provides valuable tools to further explore these questions.

The original code used to analyze the data and generate figures is available at https://github.com/Shiyang-He/MRP-Composition-and-RNA-binding-specificity and https://doi.org/10.5281/zenodo.16747756.

## Supplementary Material

gkaf829_Supplemental_Files

## Data Availability

The high-throughput sequencing data generated in this study have been deposited in GEO under accession number GSE290995. AP-MS raw files are available at PRIDE proteomics under accession number PXD061658.
