# Supplementary material for: Composition and RNA binding specificity of metazoan RNase MRP
Source: Nucleic Acids Res. 2025 Aug 27;53(16):gkaf829. doi: 10.1093/nar/gkaf829 (PMC12390755; doi:10.1093/nar/gkaf829)
Supplement: gkaf829_Supplemental_Files [file gkaf829_supplemental_files.zip › Liu_SupplMaterial.pdf]

Supplementary Material

Composition and RNA binding specificity of metazoan RNase MRP

Yuan Liu, Shiyang He, Kawon Pyo, Shanshan Zheng, Meijuan Chen, Bryony Braschi, Sihem Cheloufi, Nikolai Slavov, William F Marzluff, Jernej Murn

Supplementary Figures S1-S4

Supplementary Figure Legends S1-S4

Supplementary Table Legends S1, S2

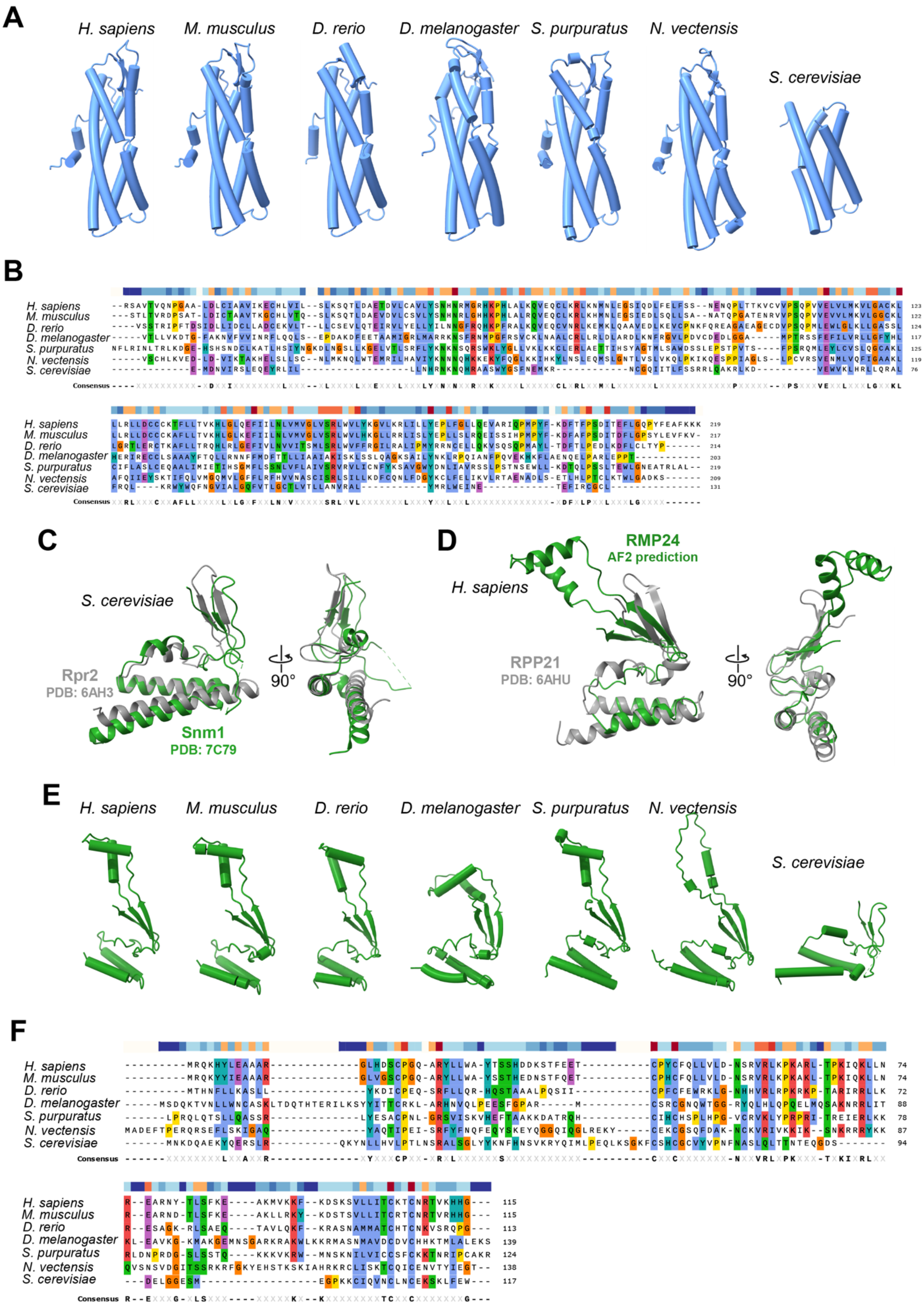
A
H. sapiens
M. musculus
D. rerio
D. melanogaster
S. purpuratus
N. vectensis
S. cerevisiae
B
H. sapiens
M. musculus
D. rerio
D. melanogaster
S. purpuratus
N. vectensis
S. cerevisiae
Consensus
C
S. cerevisiae
Rpr2
PDB: 6AH3
Snm1
PDB: 7C79
90°
D
RMP24
AF2 prediction
H. sapiens
RPP21
PDB: 6AHU
90°
E
H. sapiens
M. musculus
D. rerio
D. melanogaster
S. purpuratus
N. vectensis
S. cerevisiae
F
H. sapiens
M. musculus
D. rerio
D. melanogaster
S. purpuratus
N. vectensis
S. cerevisiae
Consensus

Supplementary Figure S1. Structural homology of known and predicted subunits of RNases P and MRP. (**A**) A representative AF2 prediction of the N-terminal helical bundle of RMP64 for each of the shown RMP64 orthologs (Homo sapiens, human; Mus musculus, house mouse; Danio rerio, zebrafish; Drosophila melanogaster, fruit fly; Stronglycentrotus purpuratus, sea urchin; Nematostella vectensis, starlet sea anemone). Cryo-EM structure of Rmp1 of Saccharomyces cerevisiae (brewer's yeast) is also shown (PDB: 7C7A). (**B**) Sequence alignment of the N-terminal helical bundle of RMP64 orthologs shown in (A) (performed with SnapGene, version 4.3.11). A conservation score is indicated above the alignment. Blue, yellow, and red colors indicate low, median, and high conservation, respectively. A consensus sequence is provided below the alignment. (**C**) Superposition of the N-terminal helices and beta-sheets of the yeast Rpr2 (PDB: 6AH3) and Snm1 (PDB: 7C79). (**D**) Superposition of the N-terminal helices and beta-sheets of the human RPP21 (PDB: 6AHU) and RMP24 (AF2 prediction). (**E**) A representative AF2 prediction of the N-terminal helices and beta-sheets of RMP24 for each of the shown RMP24 orthologs, as in (**A**). Cryo-EM structure of Snm1 of S. cerevisiae is also shown (PDB: 7C7A). (**F**) Sequence alignment of the N-terminal helices and beta-sheets of RMP24 orthologs shown in (**E**). See also (**B**).

Liu_Supplementary Figure S2

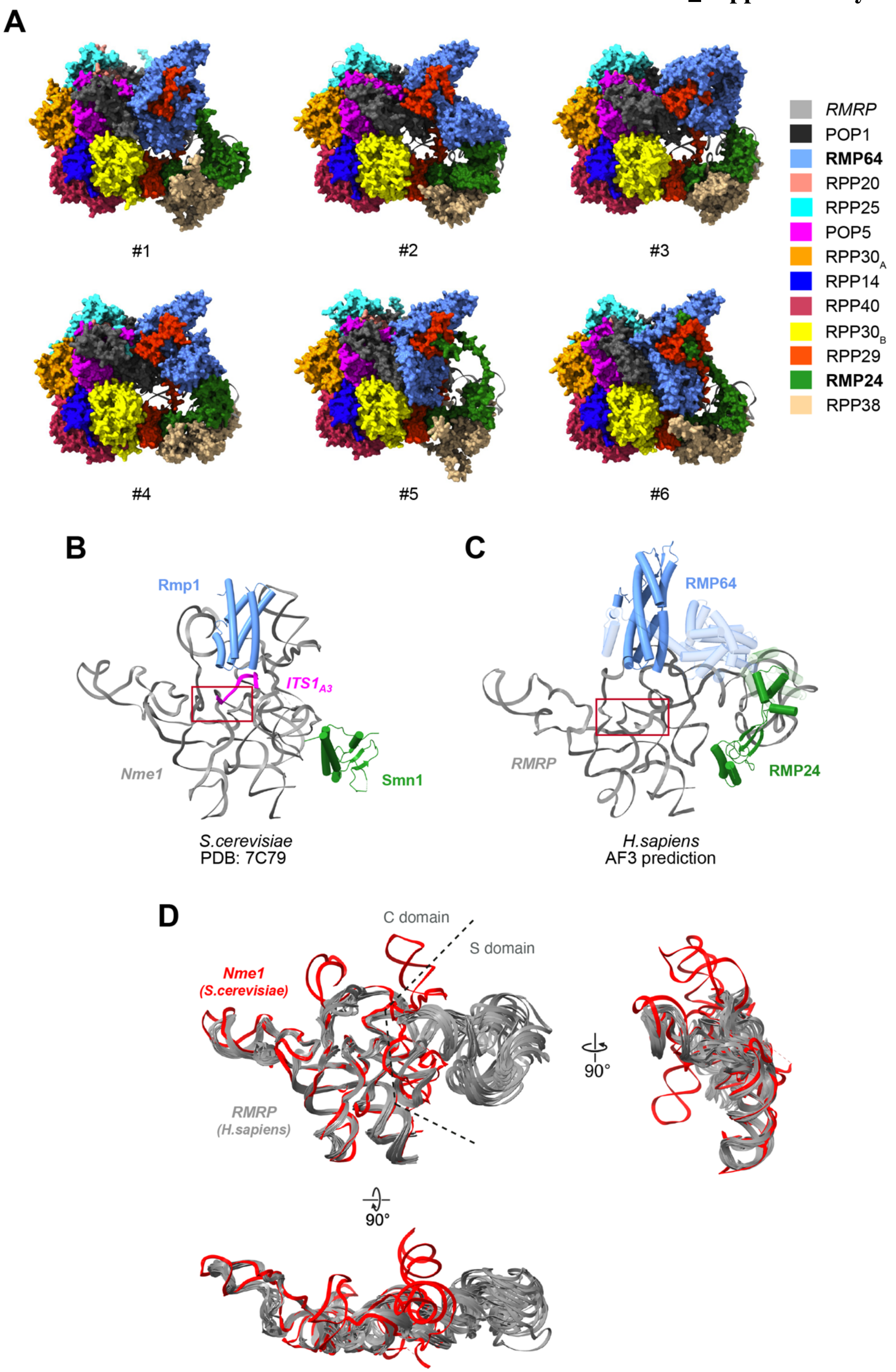

Supplementary Figure S2. Structural predictions of the human RNase MRP RNP. (**A**) Six AlphaFold3 (AF3) predictions of the human RNase MRP complex using full-length sequences of the listed subunits. Our identified protein subunits, RMP64 and RMP24, are highlighted in bold. (**B**) Cryo-EM structure of the yeast RNase MRP in complex with its RNA substrate, $ITS1_{A3}$ (PRB: 7C79). Rmp1, Snm1, *Nme1*, and $ITS1_{A3}$ are shown as visible in the structure. All other subunits are hidden. The catalytic RNA pseudoknot is highlighted with a red rectangle. (**C**) AF3 prediction #4 in (**A**), showing only *RMRP* and full-length RMP64 and RMP24. The converging protein segments from 10 independent predictions are shown opaque and the variable segments are transparent. The catalytic RNA pseudoknot is indicated with a red rectangle. (**D**) Ten AF3 predictions of the human RNase MRP and the yeast cryo-EM structure of RNase MRP (PDB: 7C79; red) were aligned on *RMRP*. Shown are only *RMRP* and *Nme1*. The gray dashed lines denote the approximate boundary between the C and S domains in *RMRP*.

Liu_Supplementary Figure S3

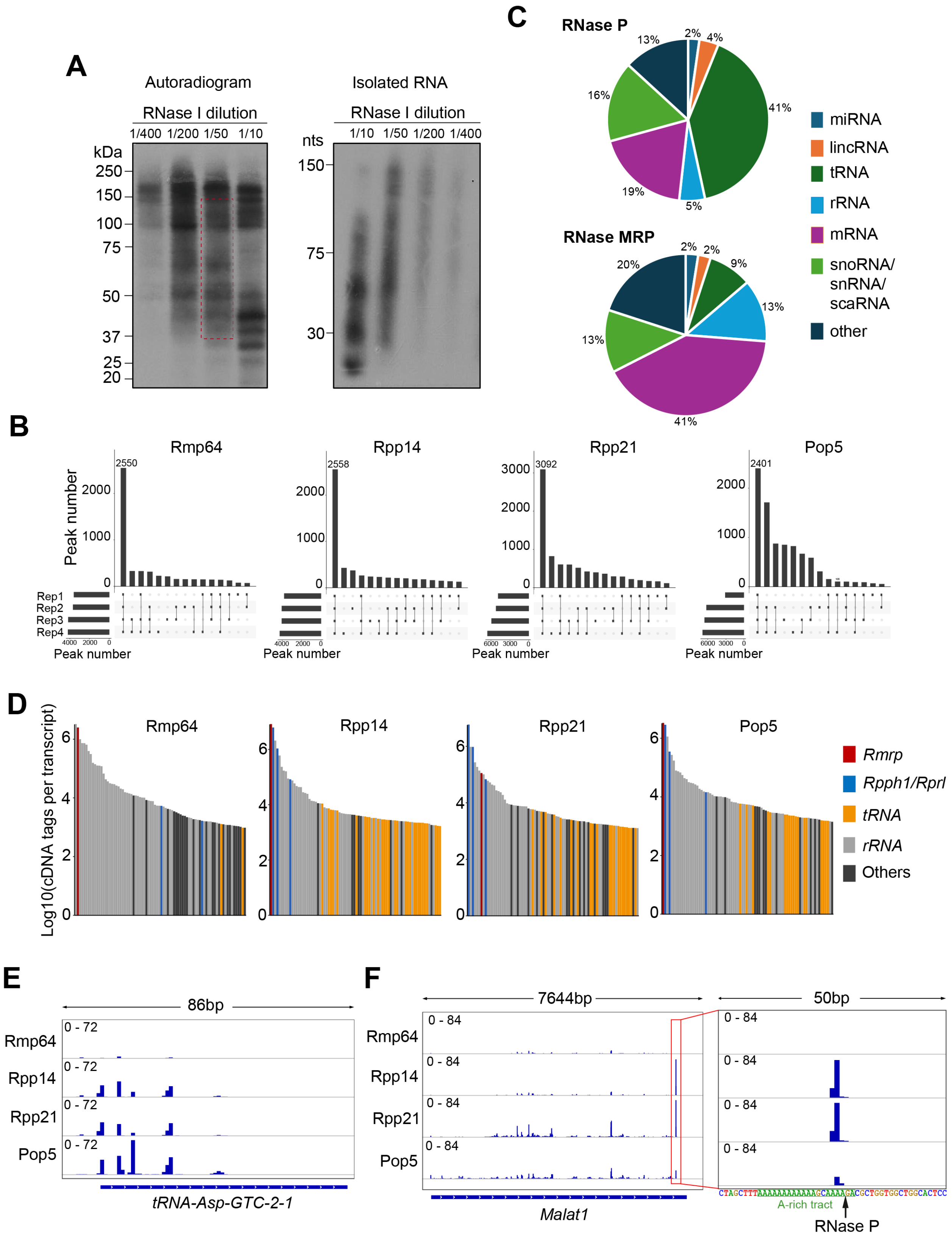

Supplementary Figure S3. RNA-binding specificity of RNases P and MRP. (**A**) Crosslinking of RNases P and MRP to RNA in mESCs. (Left) Autoradiogram of crosslinked and labeled protein/RNA complexes precipitated via Flag-tagged Rpp14. The experiment was intended for optimization of RNase I dilution. A similar pattern of radioactive bands was seen with iCLIPs of other subunits. Dashed red rectangle indicates the approximate range of crosslinked RNP complexes that were cut from the membrane and used for preparation of iCLIP libraries. (Right) Radioactively labeled RNA extracted from the crosslinked complexes on the left. (**B**) Reproducibility of the binding site (peak) calling in different iCLIP datasets. Four replicates were performed for each subunit-iCLIP experiment, and the binding sites were identified separately. Horizontal bars indicate peak numbers identified in each replicate experiment. Bar plots and dot plots indicate peak number called in different sets of replicates. (**C**) Biotype distributions of the 311 RNase P-bound and 80 RNase MRP-bound transcripts. (**D**) Top bound transcripts in each iCLIP dataset. The 80 transcripts with the highest total iCLIP cDNA tag counts (summed across all binding sites within each transcript) are shown, ordered by tag count from left to right. Gencode vM25 annotations of individual transcripts are shown. (**E**) Example of a crosslinking profile on a tRNA transcript (tRNA-Asp-GTC-2-1). (**F**) Crosslinking profile on *Malat1* shown for each iCLIP dataset. The highlighted binding peak in the left window is shown magnified on the right. Note the proximity of the peak to the annotated RNase P cleavage site within the A-rich tract (highlighted in green).

Liu_Supplementary Figure S4

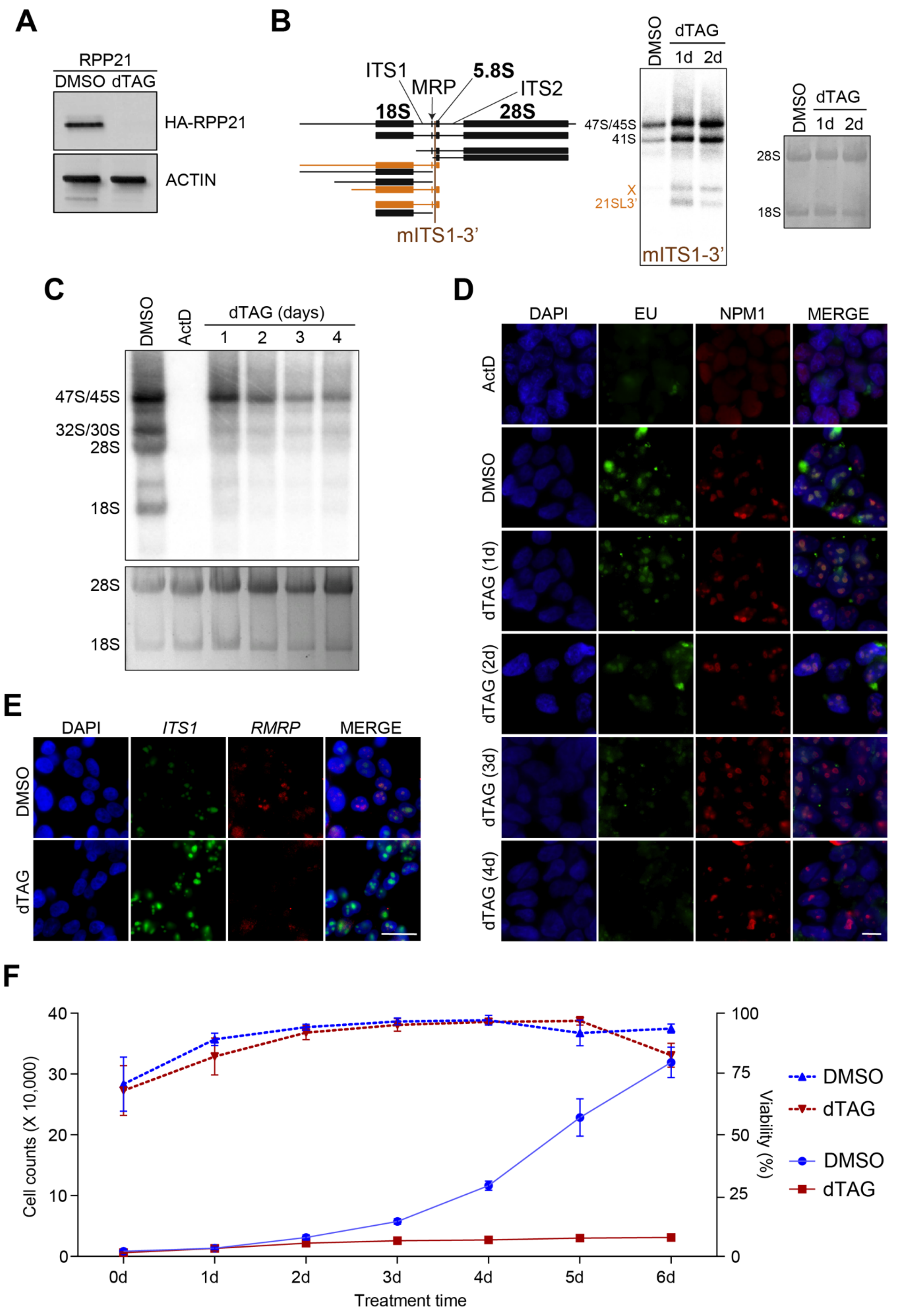

Supplementary Figure S4. Requirement of RMP64 for pre-rRNA processing and cell proliferation. (**A**) Depletion of the endogenous, HA-tagged RPP21 in our engineered HEK293T cells at 2 days of treatment with dTAG (n = 3). (**B**) Northern blot analysis in mouse embryonic stem cells (mESCs) with dTAG-inducible Rmp64 depletion. Total RNA was extracted and analyzed using a probe (mITS1–3′) targeting the 3′ end of mouse ITS1, positioned analogously to the ITS1–3′ probe used in Figure 4I. Methylene blue staining of 28S and 18S rRNAs is shown as a loading control. All RNA samples had RNA integrity numbers (RINs) of 9.3 or higher, as assessed by Agilent Bioanalyzer. (**C, D**) Analysis of the pre-rRNA transcription rate upon acute depletion of RNase MRP. (**C**) HEK293T cells engineered for dTAG-inducible degradation of RMP64 were treated with DMSO for 4 days (±30 min Actinomycin D, ActD) or with dTAG for the indicated times. Cells were labeled with [^{32}P]-*ortho*-phosphoric acid for 1 h, and total RNA was extracted, resolved by denaturing gel electrophoresis, and visualized by phosphorimaging (top). SYBR Gold staining of 18S and 28S rRNA is shown as a loading control (bottom). (**D**) Cells were treated similar as in (**C**) were pulse-labeled with 5-ethynyluridine (EU) for 30 min and processed for immunofluorescence using Click-iT Alexa Fluor 488 Imaging Kit. Nucleoli and DNA were co-stained with anti-nucleophosmin 1 (NPM1) and DAPI, respectively. Scale bar, 20 µm. (**E**) RNA FISH of ITS1 and *RMRP* at 2 days of treating RMP64-degradable HEK293T cells with DMSO or dTAG. Scale bar, 25 µm. (**F**) Growth of RMP64-degradable HEK293T cells over the indicated periods of treatment either continuously with DMSO (blue; Ctrl) or with dTAG (red). Dashed lines indicate cell viability. Data are shown as mean ± SD (n >= 3).

Supplementary Table Legends

Supplementary Table S1. RNase MRP and RNase P iCLIP peaks in mESCs.

Supplementary Table S2. Primers and oligonucleotide probes used in this study.
